# Preliminary study of central nervous system tumors’ prevalence and incidence in Isfahan Province Iran

**DOI:** 10.1186/s43046-020-00022-8

**Published:** 2020-03-19

**Authors:** Zahra Tolou-Ghamari

**Affiliations:** Isfahan Kidney Transplantation Research Center, Isfahan University of Medical Sciences, Isfahan, Iran

**Keywords:** Brain cancer, Incidence, Mortality, Prevalence

## Abstract

**Background:**

Metastatic or primary cancers of the central nervous system are a dissimilar cluster of neoplasms with different consequences and management plans. The aim of this study was to obtain prevalence and incidence of brain and other nervous system tumors.

**Results:**

In all, 423 females and 620 males were identified. For the total population the period prevalence (PP) was calculated as 20.9 per 100,000 persons. This value corresponded to a PP of 24.5 for males and 17.2 for females. The mean (SD, range) age of the patients was 52.0 (20.7, 1–99) years. In the 7% of the population studied, age was under 20 years old of life, and in the 71%, it occurred around the age of 20 to 70 years old. In both genders, incidence rate (IR) increased from 4.2 to 5.7 per 100,000 persons. The changes of IR in males versus females were 8.2 versus 3.0 from 2011 to 6.4 versus 5.0 to 2015 per 100,000 persons.

**Conclusions:**

Among the total population studied, there were 586 reported deaths. The PP in the male population was 1.4-times higher than females. There was a 35.7% increase in the IR over the study period.

## Background

Dissimilarities in the brain and central nervous system (CNS) tumor incidence have been described among countries globally [1]. According to previously published report, the incidence of brain and CNS tumors of the Western world is higher than that of the Eastern world and higher in developed countries compared with less-developed countries [2]. Differences in survival of brain and CNS tumors also exist. Studies conducted in the USA indicated that the differences in survival were mainly due to the dissimilarity in histologic category, age, sex, and management [1]. Primary brain tumors that can originate from brain cells, meninges, nerves, or glands could be a mixed cluster of benign and malignant tumors arising from the brain parenchyma and its nearby surroundings [3, 4].

Tumors of CNS involved as 25% of all tumors at the ages between 0 and 14 years, 9% of those at the ages between 15 and 24 years old, and 2% at the ages between 25 and 84 years respectively. A 5-year overall survival was reported as 10 to 15% with considerable mortality among those [5].

Previously published data on primary brain and CNS tumors associated with 11,827 patients in Korea confirmed 37.3% of meningioma followed by18.0% pituitary tumors, 12.7% gliomas, and 12.3% nerve sheath tumors. Glioblastomas accounted for 41.8% of all gliomas [6].

There seem to be some changes among the forms of brain tumor incidence in Iran and Western countries. Consequently, the prevalence of glioma reported to be about 45% of all brain tumors is rather low in comparison to the western data, but practically the same as in Southeast Asian countries [7]. The annual occurrence of CNS tumors varies from 10 to 17 cases per 100,000 persons of intracranial tumors and 1 to 2 cases per 100,000 persons for intraspinal tumors [8]. In the USA, overall incidence of malignant brain tumors was reported as 5.74 per 100,000 persons. Incidence was lowest in Southeast Asia and East Asia and was highest in Northern Europe (6.6) and Canada (6.5) per 100,000 persons respectively [9]. Study of CNS tumors for a 10-year period in Guilan/Iran showed that out of 365 cases, 80% was reported as brain tumor and the remaining revealed as spinal tumor [8].

Pediatric CNS tumors vary significantly in their histological, geographical, and sex differences during childhood and youth. A study of 67 patients comprised of 54 with brain tumors and 13 with spinal cord tumors showed that medulloblastoma was the most common brain tumor (20.3%) followed by pilocytic astrocytoma (16.6%) and glioblastoma multiforme (9.2%) [10]. Another recent published article confirmed that intramedullary spinal cord tumors comprise 1–10% of all childhood central nervous system neoplasms, with astrocytomas representing the most common subtype [11].

Due to lack of clinical data associated with brain and other nervous system tumors, this survey was intended to provide native relevant and meaningful strategy for future healthcare research in terms of pharmacotherapy or surgery management.

## Method

The Institutional Review Board approved this retrospective survey. This study was a disclosed form study by Ethics Code No. 295115. Data of the brain and other nervous system tumors from March 2011 to March 2015 were obtained from cancer registry office [11]. The Cancer Registry was located at the Deputy of health that was supported by University of Medical Sciences. Tumors of the brain and other nervous system was distinguished from the related topography codes such as C71(brain), C72 (spinal cord, cranial nerves, and other parts of central nervous system), C70 (meninges), and C47 (others; included autonomic nervous system, ganglia nerve, parasympathetic nervous system, peripheral nerve, spinal nerve, sympathetic nervous system). Microsoft Excel was used to arrange raw data before being inputted into the Statistical Package for Social Science (SPSS® version 20; IBM Corp., Armonk NY, USA) for analysis. Age, as a continuous variable, was expressed as mean ± standard deviation (SD). Variables such as gender, alive/dead, and year of the report were expressed by frequency, percentage, period prevalence (PP), and incidence rate (IR) [12].

## Results

Demographic and epidemiology characteristic of patients with brain and other nervous system tumors are shown in Table 1. There were 1043 recorded cases, in which 59.4% were males.

**Table 1:** Demographic and epidemiological data in patients with brain and other nervous system tumors

Population studied	Number	Estimated Living Cases	Estimated Death	Age (mean ± SD)	PP	Incidence rate (2011–2012)	Incidence rate (2012–2013)	Incidence rate (2013–2014)	Incidence rate (2014–2015)
Total	1043	457	586	52.0 ± 20.7	20.9	4.2	5.1	5.9	5.7
Females	423	179	244	51.8 ± 20.4	17.2	3.8	3.8	4.6	5.0
Males	620	278	342	52.2 ± 20.9	24.5	8.2	6.3	7.3	6.4

**Fig 1:**
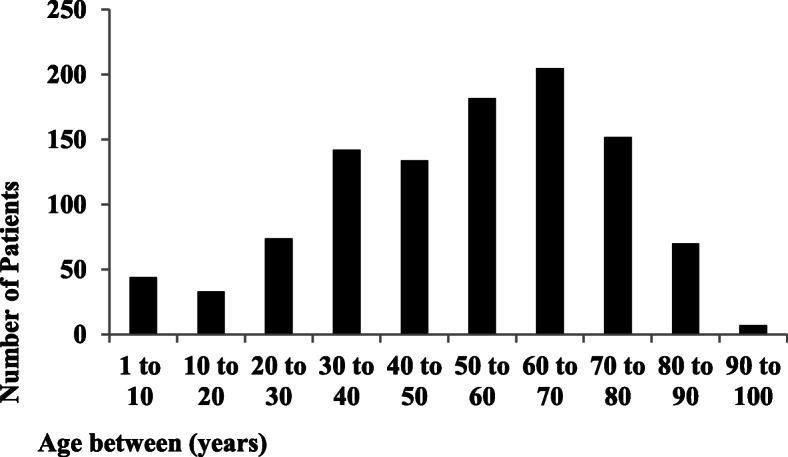
Distribution of age in total population of patients with brain and other nervous system tumors

The mean (SD; range) age was 52.0 (20.7; 1–99) years. As shown in Fig. 1, the highest incidence (71%) of age was between 20 and 70 years old. In the 7% of the population, tumor was associated to ages less than 20 years and in the 22% of the population age associated to after 70 years old.

**Fig 2:**
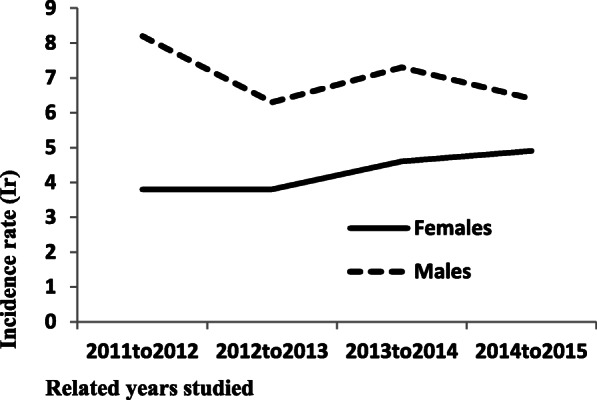
The estimated incidence of brain and other nervous system tumors related to each gender

With a total PP of 20.9 per 100,000 persons, the prevalence in males versus females was 24.5 versus 17.2 respectively. Figure 2 shows the IR for brain and other nervous system tumor between the years 2011 and 2015 in each gender. The total IR included males versus females were 4.2 (8.2 versus 3.8) for 2011–2012, 5.1 (6.3 versus 3.8) for 2012–2013, 5.9 (7.3 versus 4.6) for 2013–2014, and 5.7 (6.4 versus 5) for 2014–2015, per 100,000 persons respectively. There were 586 recorded deaths (56%) comprised of 244 females and 342 males correspondingly.

**Fig 3:**
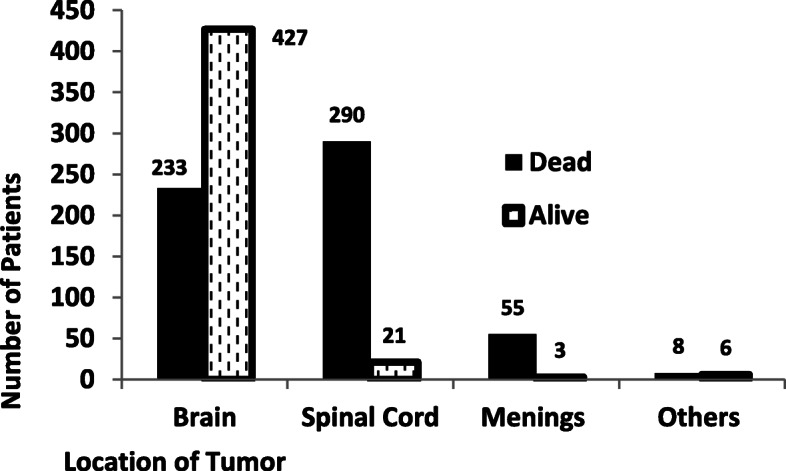
Frequency of tumors (dead/alive) according to the reported sites in total population studied

Figure 3 shows the frequency of tumors (dead versus alive) that was corresponded to: brain (*n* = 660; 233 versus 427), spinal cord (*n* = 311; 290 versus 21), meninges (58; 55 versus 3) and others (*n* = 14; 8 versus 6).

## Discussion

The incidence of CNS tumors is an important subject that could be affected of environmental and many other disparities which are inadequately understood [13]. Concerning to ethnic and racial consideration, brain tumors are less common in Asian Americans when compared with Whites in the USA and UK [1].

During this investigation, the overall PP for brain and other nervous system tumors was 20.9 per 100,000. This value is higher than the reported incidence of 15.5 cases per 100,000 in Gironde/France [13] and lower than the reported incidence of 22.4 cases per 100,000 in the USA [14].

In agreement with previous publication [14] that mentioned males not only develop more cancers but also they frequently have poorer responses to therapy as measured by event-free and overall survival, in this study, the PP for males was 1.4 times higher than females (24.5 versus 17.2 per 100,000). A study performed in the USA confirmed a higher incident for females when compared to males (24.5 versus 20 per 100,000) [14].

However, in this study there was an increase in incidence associated to both genders from 4.2 to 5.7 (35.7%) per 100,000 persons from 2011 to 2015, but related to each gender there was a 31.6% increase in females and 22% decrease in males’ tumors of the brain and other nervous system incidence.

In the 71% of the population studied in here, the highest incidence of CNS tumors was found in the age between 20 and 70 years old. This is in agreement with previous publication which confirms an incidence in most CNS tumors with age until the seventh decade [15–27].

Brain tumors are defined as neoplasms of the brain parenchyma [16, 17]; the results obtained from this study showed that the higher episode of brain and other nervous system tumors was associated to the brain (63%). Previous publication reported an overall incidence rate of all brain tumors as 10.82 per 100,000 person-years. The incidence proportion estimates were heterogeneous, even among the same tumor subtypes, and ranged from 0.051 per 100,000 (germ cell tumors) to 25.48 per 100,000 (all brain tumors) [4, 14].

As a previous publication confirmed that Iran is experiencing the increasing burden of cancers, which are currently the third leading cause of mortality in Iran [18], in this study, there was 56% recorded deaths due to tumors of the brain and other nervous system. The changing trend in mortality of cancer has also been studied in the UK. It seems that in 2011, the age-standardized mortality rate for cancer exceeded that of cardiovascular disease in both sexes [19].

## Conclusion

The report indicates that there are 13.3 million new cases of cancer in 2010, but more than half of all cancer cases and deaths worldwide are proposed to be preventable [20]. Our findings revealed that there were some differences in the period prevalence and incidence of brain and other nervous system tumors that could be associated with a combination effect of different selection practices, differences in pharmacotherapy management, changes in lifestyle, and genetic features. Additional investigations are essential to examine the factors indorsed the divergence.

## Data Availability

The datasets used and/or analyzed during the current study are available from the corresponding author on reasonable request.

## Abbreviations

SD: Standard deviation
ELC: Estimated living cases
ED: Estimated death
PP: Period prevalence
IR: Incidence rate
